# Efficacy of interventions that include diet, aerobic and resistance training components for type 2 diabetes prevention: a systematic review with meta-analysis

**DOI:** 10.1186/1479-5868-11-2

**Published:** 2014-01-15

**Authors:** Elroy J Aguiar, Philip J Morgan, Clare E Collins, Ronald C Plotnikoff, Robin Callister

**Affiliations:** 1 Priority Research Centre for Physical Activity and Nutrition, University of Newcastle, Callaghan Campus, University Dr, Callaghan, NSW 2308, Australia; 2 School of Biomedical Sciences and Pharmacy, Faculty of Health, University of Newcastle, Callaghan Campus, University Dr, Callaghan, NSW 2308, Australia; 3 School of Education, Faculty of Education and Arts, University of Newcastle, Callaghan Campus, University Dr, Callaghan, NSW 2308, Australia; 4 School of Health Sciences, Faculty of Health, University of Newcastle, Callaghan Campus, University Dr, Callaghan, NSW 2308, Australia

**Keywords:** Prediabetes, Diabetes, Obesity, Lifestyle, Weight loss, Diet, Exercise, Resistance training

## Abstract

Current recommendations for the prevention of type 2 diabetes advise modification of diet and exercise behaviors including both aerobic and resistance training. However, the efficacy of multi-component interventions involving a combination of these three components has not been established. The aims of this review were to systematically review and meta-analyze the evidence on multi-component (diet + aerobic exercise + resistance training) lifestyle interventions for type 2 diabetes prevention. Eight electronic databases (Medline, Embase, SportDiscus, Web of Science, CINAHL, Informit health collection, Cochrane library and Scopus) were searched up to June 2013. Eligible studies 1) recruited prediabetic adults or individuals at risk of type 2 diabetes; 2) conducted diet and exercise [including both physical activity/aerobic *and* resistance training] programs; and 3) reported weight and plasma glucose outcomes. In total, 23 articles from eight studies were eligible including five randomized controlled trials, one quasi-experimental, one two-group comparison and one single-group pre-post study. Four studies had a low risk of bias (score ≥ 6/10). Median intervention length was 12 months (range 4–48 months) with a follow-up of 18 months (range 6.5 - 48 months). The diet and exercise interventions varied slightly in terms of their specific prescriptions. Meta-analysis favored interventions over controls for weight loss (-3.79 kg [-6.13, -1.46; 95% CI], Z = 3.19, P = 0.001) and fasting plasma glucose (-0.13 mmol.L^-1^ [-0.24, -0.02; 95% CI], Z = 2.42, P = 0.02). Diabetes incidence was only reported in two studies, with reductions of 58% and 56% versus control groups. In summary, multi-component lifestyle type 2 diabetes prevention interventions that include diet and both aerobic and resistance exercise training are modestly effective in inducing weight loss and improving impaired fasting glucose, glucose tolerance, dietary and exercise outcomes in at risk and prediabetic adult populations. These results support the current exercise guidelines for the inclusion of resistance training in type 2 diabetes prevention, however there remains a need for more rigorous studies, with long-term follow-up evaluating program efficacy, muscular fitness outcomes, diabetes incidence and risk reduction.

## Introduction

Type 2 diabetes mellitus (T2DM) is one of the fastest growing non-communicable diseases worldwide [1,2]. Recommendations for T2DM prevention include maintaining a healthy weight, consuming a healthy diet, and participation in exercise. Most T2DM prevention programs have recommended aerobic (cardio-respiratory) activities [3] with strong evidence supporting this approach. Large-scale prevention studies such as the Diabetes Prevention Program (DPP) [4] reported reductions in T2DM incidence of up to 58% [5] and improvements in risk factors such as weight and insulin sensitivity.

More recently, resistance training (RT) has been included in guidelines for T2DM based on evidence established over the last decade, which demonstrates benefits from RT including improved fasting plasma glucose (FPG) [6-11], glycosylated haemoglobin (HbA_1C_) [8-16], insulin sensitivity [8,14] and the maintenance of fat free mass during energy restriction for weight loss [17,18]. Current guidelines for T2DM prevention and management [3,19] recommend at least 150 min per week of moderate-vigorous aerobic activity and an additional two (ideally three) RT sessions per week (at least 60 min). Studies have reported that the combination of aerobic plus RT has additive benefits on glucose control [16,20,21] and can achieve greater reductions in T2DM incidence [22,23] than the use of a single exercise modality. However, multi-component (diet + aerobic exercise + RT) lifestyle interventions have the potential to become excessively burdensome, which could compromise program adherence. Further, the long-term efficacy of multi-component programs remains unclear.

Therefore, the aim of this systematic review was to summarize the evidence of the efficacy of lifestyle interventions that include diet + aerobic exercise + RT components in at risk or prediabetic populations. Specifically, this review assesses the effects of these interventions on weight change, glucose regulation, and diet and exercise outcomes. A secondary aim was to conduct a meta-analysis of the impact on weight and FPG. Addressing these aims is necessary to validate the evidence supporting current dietary and exercise guidelines for T2DM prevention.

## Methods

The Preferred Reporting Items for Systematic reviews and Meta-Analyses (PRISMA) statement [24] guided the conduct and reporting of this review.

### Information sources

A systematic literature search was conducted using electronic databases (Medline, Embase, SportDiscus, ISI Web of Knowledge [Web of Science], CINAHL, Informit health collection, Cochrane library, Scopus) until June 2013. No limit was placed on publication date. The search strategy included the use of terms in three broad categories: (i) population; (ii) intervention; and (iii) study type. The search terms list included the following items: pre-diabetic OR prediabetic OR pre-diabetes OR prediabetes OR glucose intolerance OR impaired glucose tolerance OR impaired fasting glucose AND exercise OR resistance training OR weight lifting OR aerobic training OR diet OR lifestyle OR life-style AND randomized controlled trial OR randomised controlled trial OR controlled clinical trial OR randomized OR randomised OR randomly OR trial OR groups OR intervention OR study OR program. Reference lists of included studies and key reviews in the area were also manually searched for additional articles.

### Eligibility criteria

Studies were included if they: (i) targeted T2DM prevention in at risk or prediabetic adults (>18 years); (ii) employed a lifestyle diet and exercise intervention including both aerobic *and* RT; and (iii) reported weight and plasma glucose. All study designs were considered. Studies were excluded if they: (i) recruited individuals with T2DM; (ii) recruited individuals diagnosed with severe medical problems unrelated to prediabetes or from other special populations (e.g., mental illness, polycystic ovarian syndrome, gestational diabetes); (iii) used drug therapy or surgical procedures as part of the intervention.

### Study selection

After duplicate deletion, one author (EA) screened all articles based on title and abstracts for preliminary inclusion; then screened remaining articles by full text based on inclusion criteria. In cases where there was uncertainty, a second reviewer (RC) assessed the article and consensus was reached by discussion.

### Data collection process and data items

Characteristics and results of studies were extracted by one author (EA). Studies with multiple published articles were reported as a single group. For meta-analyses, final mean and standard deviation (SD) or change in mean and SD were extracted for weight (kg) and FPG (mmol.L^-1^). In some studies, the required statistics for meta-analysis were not reported. If available, other statistics e.g., 95% confidence interval (CI) or standard error (SE) were converted to the required form according to the calculations outlined in the Cochrane Handbook for Systematic Reviews of Interventions (Section 7.7 and 16.1.3.2) [25].

### Risk of bias in individual studies

Risk of bias for individual studies was assessed for randomized trials using a 10-item quality checklist adapted from the Consolidated Standards of Reporting Trials (CONSORT) statement [26]. The 10-item scale and explanations of the scoring for each item are available (see Additional file 1). Each item was scored with a ‘1’ for ‘yes’ or ‘0’ for ‘no’. Inter-rater reliability was calculated on a dichotomous scale using percentage agreement and Cohen’s κ. Un-weighted sum totals were calculated for each study. Based on a dichotomy used in recent reviews [27,28] studies were classified as having a low (score ≥ 6) or high risk of bias (score ≤ 5). Two authors (EA and RC) assessed the risk of bias in the individual studies that met the inclusion criteria. In the case of disagreement, discussion took place until consensus was reached.

### Summary measures and synthesis of results

The primary outcomes for the review were the between group difference in means for weight (kg) and FPG (mmol.L^-1^). Secondary outcomes included 2-h oral glucose tolerance test (OGTT, mmol.L^-1^) and HbA_1C_%, dietary outcomes (e.g., macronutrient composition) and exercise outcomes (e.g., physical activity, aerobic and muscular fitness). Meta-analyses for weight and FPG were conducted for eligible randomized controlled trials (RCT). Results were pooled in separate meta-analyses using RevMan 5.1.4 for Mac OS X. All data were continuous and reported on the same scale for weight (kg) and FPG (mmol.L^-1^). Heterogeneity of studies included for meta-analysis was determined using Chi^2^ and I^2^ statistics. A significance level of P < 0.10 for the Chi^2^ test and an I^2^ greater than 50% indicated substantial heterogeneity [24]. The fixed-effects model was used for homogenous samples and the random-effects model was used where heterogeneity was present. The aggregate result was calculated as the weighted mean difference (WMD) between interventions and controls. Meta-analysis was deemed inappropriate for variables where results from fewer than three studies were available.

## Results

### Study selection

**Figure 1:**
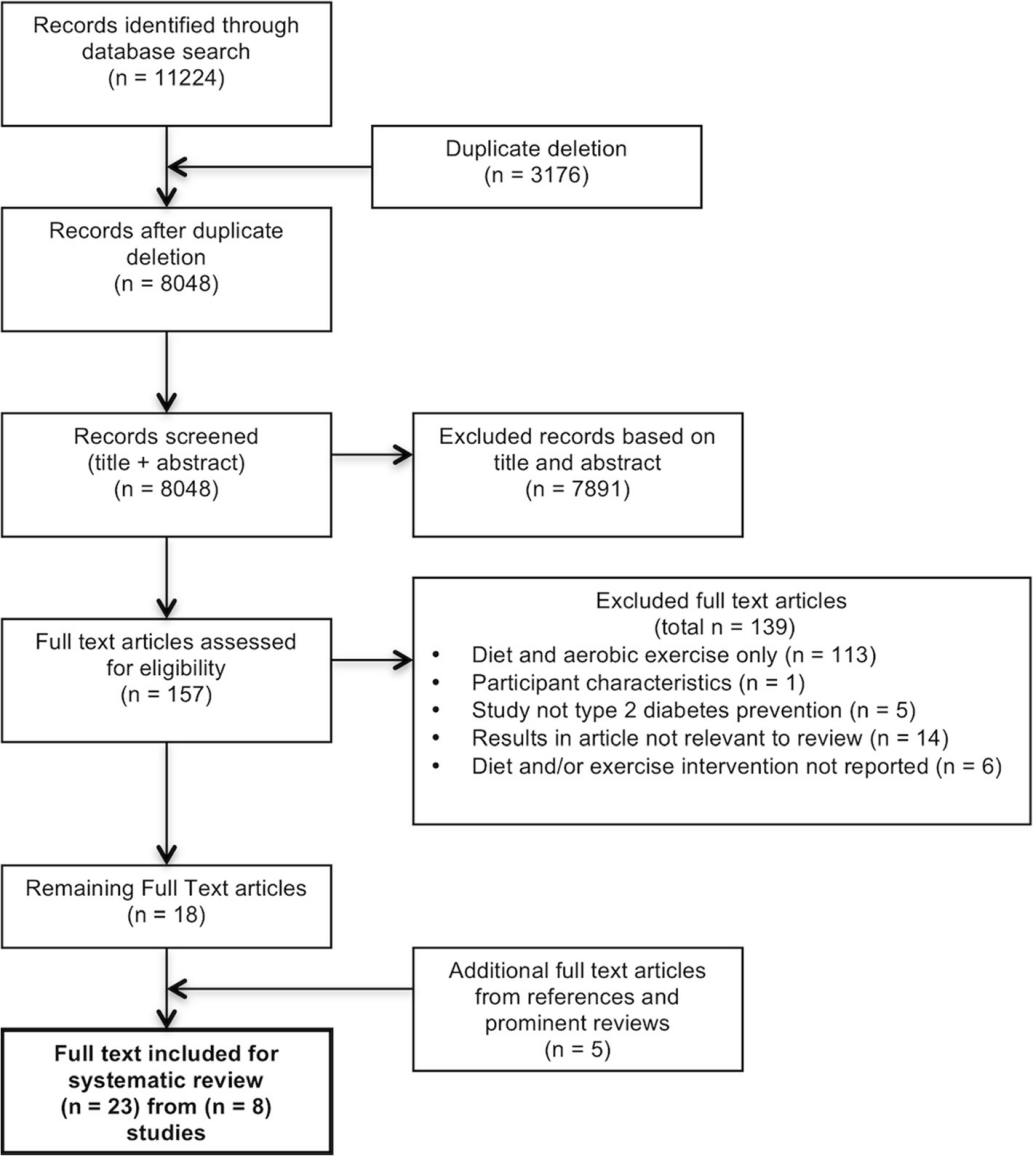
**PRISMA flow diagram of study selection.** Describes the process of article searching, exclusion (with reasons) and selection of studies of systematic review and meta-analyses.

After duplicate deletion, 8048 original articles were identified (Figure 1). After title/abstract screening and further full-text screening, 23 articles arising from eight studies were deemed eligible. Of these, four studies were eligible for meta-analysis of weight and five studies for meta-analysis of FPG.

### Study characteristics

Of the eight included studies, two were conducted in the United States [29-32], one in New Zealand [33,34], Austria [35], the Netherlands [36-40], Australia [41], Finland [42-49] and the United Kingdom [50,51]. Characteristics of these studies are presented in Additional file 2. Five studies [29-31,33,34,36-40,42-51] used an RCT design, with the remaining studies employing quasi-experimental [41] two-group comparison [35] or single-group pre-post designs [32]. Three studies specifically recruited impaired glucose tolerance (IGT) participants [33,42-51], one study recruited impaired fasting glucose (IFG) participants [35], one study recruited both IGT and IFG participants [32], and three studies recruited an ‘at risk for prediabetes’ population [29-31,36-41]. The collective sample size of the studies at baseline was 1050 participants, with females comprising 62% of the sample. Mean (± SD) age was 54.5 ± 9.7 years. Five studies [33-40,42-51] used an individual face-to-face mode as the primary means of intervention delivery, while three studies [29-32,41] used a group face-to-face mode. All studies conducted supervised individual and group exercise programs for some period of the intervention (Additional file 2). One study had an initial one-week period with a physical therapist and thereafter the exercise program was self-driven [32]. Most studies used gym facilities, however one study used an unsupervised home-based RT component for one of their intervention groups [41].

Median intervention length was 12 months (range 4–48 months) with a follow-up of 18 months (range 6.5 - 48 months). Details of the diet and exercise interventions are reported in Additional file 2. Briefly, participants were advised to perform aerobic exercise for an average of 5.0 ± 1.5 days.wk^-1^ (mean ± SD), with an average duration of 157.5 ± 44.4 min.wk^-1^ and to perform RT for an average of 2.3 ± 0.7 days.wk^-1^ for an average duration of 90.0 ± 24.5 min.wk^-1^. Five studies prescribed energy restriction for weight loss and seven studies prescribed a specific dietary macronutrient profile.

### Risk of bias within studies

The 10-item risk of bias analysis results for seven of the eight included studies are presented in Table 1. Bersoux et al. [32] was excluded as it was a non-randomized trial. Inter-rater reliability demonstrated a high initial level of agreement across all risk of bias items (percentage agreement 100%, Cohen’s κ = 1), with no further discussion required. In total, three studies were classified as having a high risk of bias (score ≤ 5) [35,41,50,51] and four studies as having a low risk of bias (score ≥ 6) [29,30,33,34,36-40,42-49].

**Table 1:** Risk of bias analysis for randomized studies

**Author, year, Study Name (SN)**	**i) Did the study report a power calculation and was the study adequately powered to detect intervention effects?**	**ii) Was the randomization procedure adequately described and carried out?**	**iii) Did the study include a control group? (randomized participants not a comparison group)**	**iv) Did the study present baseline characteristics separately for treatment groups?**	**v) Did the study analyses account for potential differences at baseline?**	**vi) Were the assessors blinded to treatment allocation at baseline and post-test?**	**vii) Did the study have a dropout of <20% (<6 month follow-up) or <30% (>6 month follow-up for the primary outcome of weight**	**vii) Did the study use an intention to treat analysis?**	**ix) Did the study report summary results for each group?**	**x) Did the study report precision estimates (eg 95% confidence interval) and/or effect sizes?**	**Total**
**Author** Burtscher et al. [35]	0	0	0	1	1	0	0	0	1	0	3
**Authors** Lindstrom et al. [44], (Eriksson et al. [42] (Laaksonen et al. [43], Lindstrom et al. [45], Lindstrom et al. [46], Tuomilehto et al. [47], Uusitupa et al. [48], Uusitupa et al. [49]) **SN:** Finnish DPS	0	1	1	1	1	0	1	1	1	1	8
**Author** McAuley et al. [34], (Dale et al. [33])	0	1	1	1	1	1	1	1	1	1	9
**Authors** Page et al. [50] & Page et al. [51]	0	1	1	1	0	0	1	0	1	0	5
**Author ** Payne et al. [41]	0	0	0	0	1	0	1	1	0	1	4
**Authors** Roumen et al. [39], (Corpeleijn et al. [36], Mensink et al. [37], Mensink [38], Roumen et al. [40]) **SN:** The SLIM study	1	1	1	1	1	1	1	1	1	1	10
**Authors** Villareal [29,31], (Villareal et al. [30], Villareal [29,31])	0	1	1	1	1	1	1	1	1	1	9

### Results of included studies

A summary of results is presented in Additional file 3. A brief description of results is presented below. Results are presented as change in mean from baseline to end of intervention or as pre-post intervention. Follow-up results (where available) are presented in Additional file 3.

#### Weight change

Seven of the eight studies reported a reduction in weight (kg) for the intervention group at the end of their respective interventions and four of the five RCTs reported significant weight loss for the intervention group compared to controls (Additional file 3). The largest weight loss (-8.2 ± 5.7 kg) was reported by Villareal et al. [29-31] after 26 weeks of intervention. The Finnish DPS [42-49] and SLIM studies [36-40] reported a reduction in weight for the intervention compared to controls (INT -3.5 ± 5.1 vs CON -0.9 ± 5.4 kg, P < 0.001 and INT -1.08 ± 4.30 vs CON 0.16 ± 4.91 kg, P = 0.045, respectively) after three years, demonstrating that small-moderate long-term weight loss is achievable in this population.

#### Glucose regulation

FPG was reported in all eight studies (Additional file 3). Only two of the five RCTs reported significant differences between the intervention and control groups. Villareal et al. [29-31] reported the intervention group had a reduction in FPG at 26 weeks whereas the control group increased (P < 0.05). In the SLIM study [36-40], FPG increased in both groups after three years relative to baseline, however the difference between groups was significant (P = 0.04). Payne et al. [41] and Burtscher et al. [35] reported significant within group pre-post reductions in FPG (P < 0.001 and P < 0.05, respectively) at 12 months. Two-hour OGTT was reported in five studies (Additional file 3). Villareal et al. [29-31] reported a reduction in 2-h OGTT at 26 weeks for the intervention group whereas the control group increased (P < 0.05). Bersoux et al. [32] and Payne et al. [41] reported within group pre-post reductions (P = 0.04 and P = 0.011, respectively) at 6 and 12 months, respectively. HbA_1C_ was reported in two RCTs (Additional file 3). The Finish DPS [42-49] intervention group had a significant reduction compared to controls (P = 0.002) at three years, whereas the SLIM study [36-40] reported no difference between groups (P = 0.838) at three years.

#### Exercise outcomes

Physical activity and/or physical fitness outcomes were questionnaire [29-31,41-49] or exercise testing [29-31,33-40,50,51] based (Additional file 3). Aerobic exercise outcomes were reported in five of the eight studies. McAuley et al. [34] and Dale et al. [33] predicted aerobic capacity (VO_2max_) from performance in a sub-maximal walking test (modified Bruce protocol). The intensive intervention group improved compared to controls (P = 0.02) whereas the modest intervention group did not (P = 0.94). In the SLIM study [36-40] aerobic capacity (VO_2max_) was assessed using an incremental exhaustive cycle ergometer test. At three years, the intervention group improved their aerobic capacity compared to controls (P = 0.009). At 26 weeks, Villareal et al. [29-31] reported the intervention group VO_2peak_ had improved compared to the controls (P = 0.02). Page et al. [50,51] measured aerobic capacity (VO_2max_) using maximal cycle ergometry. The ‘healthy living’ intervention group improved aerobic capacity (P < 0.05) whereas the controls did not change (P value not reported) at six months. Burtscher et al. [35] reported a pre-post increase in maximum metabolic equivalents (METs_max_) for their ‘counseling + supervised exercise’ group after one year, which differed (P = 0.01) to the ‘counseling only’ group.

Only one of the eight studies measured improvements in muscular strength or performance (Additional file 3). Villareal et al. [29-31] reported significant improvements in knee extension (P = 0.04) and knee flexion (P = 0.008) for the intervention group versus controls after 26 weeks.

No studies used objective measures (e.g., pedometers or accelerometers) to assess physical activity. Self-reported physical activity was presented in three studies (Additional file 3). The Finnish DPS [42-49] reported no difference in mean total leisure time physical activity (P = 0.2415), but moderate-vigorous leisure time physical activity increased in the intervention group compared with controls (P = 0.006) at three years (validated self-report questionnaire). Burtscher et al. [35] reported that duration of physical activity (min.wk^-1^ from log books) during the last three months of intervention in the supervised exercise group was almost double that of the counseling only group (P value not reported). Payne et al. [41] reported pre-post intervention increases in physical activity weighted min.wk^-1^ (P = 0.007) and sessions.wk^-1^ (P = 0.004) after one year (validated self-report questionnaire).

#### Dietary outcomes

Dietary composition was assessed in six of the eight studies. Total energy intake (E) expressed as kilocalories (kcal) or kilojoules (kJ) per day was reported in five studies (Additional file 3). The Finnish DPS [42-49] reported reductions favoring intervention over controls at three years (P = 0.007). McAuley et al. [34] and Dale et al. [33] reported a reduction in energy intake for modest and intensive intervention groups at four months, however only the modest group (P = 0.005) was significantly different to controls. Significant within group pre-post reductions for energy intake were reported by Page et al. [50,51] (P < 0.01) at six months and Payne et al. [41] (P < 0.001) at 12 months. Results for intervention effects on macronutrient composition are provided in Additional file 3.

### Type 2 diabetes incidence

T2DM incidence was only reported in two studies. The Finnish DPS [42-49] reported the cumulative incidence of T2DM after four years was 58% lower in the intervention group than controls [47]. The SLIM study [36-40] reported cumulative incidence for T2DM after three years of 18% (11/61) for intervention and 32% (19/60) for the controls (56% lower for the intervention compared to control).

### Synthesis of results

Meta-analysis of RCTs with outcomes for weight and FPG were conducted. Funnel plots to assess publication bias were not generated as fewer than 10 interventions were included in the meta-analysis [25].

#### Weight change

**Figure 2:**
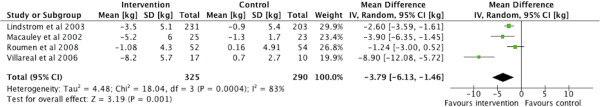
**Forrest plot – weight loss (kg).** Meta-analysis forest plot comparison of weight loss (kg) in randomized controlled trials (intervention vs control) at the last reported assessment. **Tau**^**2**^ – Tau square test; **Chi**^**2**^ = Chi square test; **df** = degrees of freedom; **I**^**2**^ = I-squared statistic; **IV** = inverse variance; **Z** = Z-test.

In total, 325 intervention and 290 control participants (total 644) from four studies were included. The interventions were statistically heterogeneous (χ^2^ = 18.04, d.f. = 3, P < 0.001, I^2^ = 83%), so the random effects model was used. Meta-analysis (Figure 2) revealed a significant reduction in weight favoring the interventions over controls at the last reported assessment (WMD -3.79 kg [-6.13, -1.46; 95% CI], Z = 3.19, P = 0.001). The time frame of assessments varied from four to 36 months.

#### Fasting plasma glucose

**Figure 3:**
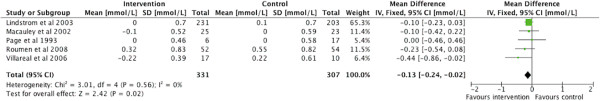
**Forrest plot – fasting plasma glucose (mmol.L**^**-1**^**).** Meta-analysis forest plot comparison of change in fasting plasma glucose in randomized controlled trials (intervention vs control) at the last reported assessment. **Chi**^**2**^ = Chi square test; **df** = degrees of freedom; **I**^**2**^ = I-squared statistic; **IV** = inverse variance; **Z** = Z-test.

In total, 331 intervention and 307 control participants (total 667) from five studies were included. The interventions were statistically homogenous (χ^2^ = 3.01, d.f. = 4, P = 0.56, I^2^ = 0%), so the fixed effects model was used. Meta-analysis (Figure 3) revealed a significant reduction in FPG favoring interventions over controls at the last reported assessment (WMD -0.13 mmol.L^-1^ [-0.24, -0.02; 95% CI], Z = 2.42, P = 0.02), with the time frame from four to 36 months.

## Discussion

This systematic review found that multi-component lifestyle interventions incorporating diet + aerobic exercise + RT conducted in at risk or prediabetic adult populations were efficacious for inducing modest weight loss and eliciting small improvements in glycemic control, together with improvements in aerobic fitness and dietary intake. The impact of interventions on muscular fitness and physical activity were not consistently reported, making it difficult to determine the contributions of these components towards improvements in glucose regulation.

### Weight change and glucose regulation

All interventions in this review and the meta-analysis found significant weight loss compared to controls. Importantly the DPP identified weight loss as the dominant predictor of their 58% reduction in T2DM incidence [52]. However, the effects on glucose regulation were less consistent. Meta-analysis found a small but significant reduction that would be of clinical importance in those with borderline prediabetes. The baseline FPG mean of the combined study population in the meta-analysis (5.6 mmol.L^-1^) was at the lower limit of the prediabetes range (5.6 - 6.9 mmol.L^-1^) [53]. This suggests that scope to improve further was limited in these cohorts, a circumstance which may be common amongst prediabetic individuals who present in clinical settings. Furthermore, the small magnitude of change observed in the meta-analysis for FPG was heavily influenced by the results of the Finnish DPS, which received a 65% weighting due to its large sample size. The Finnish DPS excluded participants from the study after diagnosis of T2DM. As the majority of those developing T2DM belonged to the control group, this introduces bias, which underestimates the FPG of the control group, leading to an attenuation of the difference between the groups.

### Exercise programs and measurement of related outcomes

The reporting of exercise programs was inconsistent between studies and most studies provided only general descriptions of their exercise programs. For example, “The supervised exercise group has additionally been offered supervised, progressive, individually tailored aerobic exercise programs and circuit-type resistance training sessions for 1 hour twice a week” [35]. This makes it difficult to determine the specific modes of RT exercises that were performed (e.g., body weight, free weights, isometric exercises, isokinetic exercises, resistance band) and the volume (load, repetitions and sets) prescribed. Future studies are recommended to provide more comprehensive descriptions of the exercise programs. Most studies provided supervised individual or group exercise sessions; only one study included a home-based exercise component [41]. This has implications for the feasibility, practicalities and dissemination costs of these programs into community and health-care settings, as few health care systems can afford to provide supervision of exercise programs by qualified personnel.

Measurement of exercise-related outcomes was also inconsistent between studies. No studies used objective measures (e.g., pedometers or accelerometers) to assess physical activity, which is a major limitation in existing studies. Physical activity levels as measured by self-report improved in intervention groups versus controls groups [42-49]. Aerobic exercise tests to measure or predict VO_2max_ were the most widely used fitness indicator, and improvements in aerobic fitness in intervention groups were generally observed [29-31,33,34,36-40]. Only one study measured improvements in muscular strength [29-31], assessing only lower body limb strength using an isokinetic dynamometer. Without evaluation of muscular performance (including upper and lower body muscle groups) it is difficult to determine whether the RT program was adhered to or whether the addition of RT in multi-component programs contributes to improvements in muscular fitness and glycemic control in prediabetes populations, as has been shown in adults with T2DM [54]. Future studies should provide comprehensive and objective evaluation of the impact on aerobic and muscular fitness.

### Type 2 diabetes incidence

A reduction in T2DM incidence is the goal for all T2DM prevention programs. Of the studies reviewed, incidence of T2DM was only reported in the Finnish DPS and SLIM studies (up to 58% reduction in T2DM incidence). This finding is of great interest, particularly since the US DPP, which did not prescribe RT as part of their physical activity recommendations, also reported a 58% reduction in diabetes incidence (after 2.8 years) [5]. This suggests that multi-component T2DM prevention programs that include RT are effective, but whether RT provides benefits additional to dietary and aerobic components requires further investigation.

### Features of effective interventions

Study design and intervention components were heterogeneous amongst the included studies, which may account for some of the variation observed in the outcomes assessed. Design characteristics of studies that achieved significant changes for weight loss and FPG [29-31,33,36-39,41] included: face-to-face intervention delivery mode (individual and/or group), an average of eight contacts per month (including face to face sessions, emails and phone calls), and a minimum of six (preferably 12) months of follow up. Lifestyle intervention characteristics included: 150–210 minutes (3–5 sessions) of aerobic exercise per week; 60–120 minutes (1–3 sessions) of RT per week; recommendations for a specified macronutrient diet profile, energy restriction for weight loss and setting a weight loss goal of 5-10%.

### Sex differences in lifestyle programs

Of the studies reviewed, 62% of participants were female. Since there is no reported global difference in gender distribution for diabetes [1], this may indicate that women are more likely to participate in diabetes prevention trials. None of the studies targeted a specific sex or reported their results by sex. Whether males and females benefit equally from these multi-component interventions is not known, but future studies should report their results by sex to reveal any differences that may exist. A recent systematic review [55] argued that sex-specific design features may be important influences on the effectiveness of lifestyle interventions.

### Strengths and limitations

This is the first review to synthesize the evidence of multi-component interventions including diet, aerobic exercise and RT for the prevention of type 2 diabetes. It adhered to the PRISMA statement for the reporting of systematic reviews and meta-analyses; a comprehensive search strategy was performed across multiple databases with no date restrictions; high agreement levels for quality assessments were achieved; and detailed data extraction was performed to allow for comparisons between studies.

The review also has some limitations. Meta-analyses for weight and FPG were based on a small number of studies and the meta-analysis for weight was statistically heterogeneous. The sample for the meta-analyses consisted of 62% females, which introduces a sex bias. Furthermore the mean age of participants was 54.5 ± 9.7 years and only one study targeted older individuals (>65) [29-31]. This limits the generalizability of the results particularly for older individuals and highlights an evidence gap in the field. Regular resistance training may result in gains or maintenance of muscle mass; consequently weight loss as an outcome by itself would be confounded by the inability to discriminate between loss of fat mass and gains in fat free mass. Future studies need to include more comprehensive assessments of body composition. For the aforementioned reasons results from the original studies and the synthesis of results presented here must be interpreted with caution. Finally, T2DM prevention studies that employed diet + aerobic exercise, but not RT were not eligible, including the highly successful US DPP.

### Direction for future research

This review has highlighted the need for high quality long-term RCTs that assess multi-component lifestyle prevention programs for T2DM. Systematic investigation of the benefits of each additional component (diet, aerobic, RT, physical activity) of multi-component lifestyle interventions is also required to provide further support for the current recommendations for T2DM prevention. Future studies should report intervention component adherence and use objective measures to detect changes in muscular fitness, aerobic capacity and physical activity. More comprehensive measures of body composition (e.g., waist circumference, dual x-ray absorptiometry or bioimpedance analysis) should be utilised to determine changes in body composition as a result of multi-component T2DM prevention programs including RT. Studies exploring interventions tailored specifically for men or women are required to determine any impact on recruitment, retention and efficacy.

## Conclusions

Multi-component lifestyle interventions to prevent T2DM, which include a dietary intervention and both aerobic and resistance exercise training, are modestly effective in inducing weight loss, improving impaired fasting glucose, improving glucose tolerance and improving dietary and exercise outcomes in at risk and prediabetic adult populations. These results support the current exercise guidelines for the inclusion of RT in T2DM prevention. Further research is required to determine the long-term efficacy of multi-component interventions on T2DM prevention and changes in biomarkers of risk and the specific contributions of each intervention component to these outcomes.

## Competing interests

The authors declare that they have no competing interests.

## Authors’ contributions

EA was chiefly responsible for data acquisition, analysis and interpretation, as well as drafting the manuscript. All other authors (PM, CC, RP, RC) were responsible for conception and design, data interpretation, revisions, and final approval of the article.

## Supplementary Material

Additional file 1 **Risk of bias assessment explanation.** 10-item risk of bias assessment criteria and an explanation of the scoring details for each item. Click here for file

Additional file 2 Characteristics of included studies. Click here for file

Additional file 3 Results of included studies. Click here for file
