# Supplementary material for: Efficacy of interventions that include diet, aerobic and resistance training components for type 2 diabetes prevention: a systematic review with meta-analysis
Source: Int J Behav Nutr Phys Act. 2014 Jan 15;11:2. doi: 10.1186/1479-5868-11-2 (PMC3898566; doi:10.1186/1479-5868-11-2)
Supplement: Additional file 1 — Risk of bias assessment explanation. 10-item risk of bias assessment criteria and an explanation of the scoring details for each item. [file 1479-5868-11-2-S1.pdf]

Additional file 1. Risk of bias assessment explanation

10-item risk of bias assessment criteria and an explanation of the scoring details for each item

Criteria	Yes	No
i) Did the study report a power calculation and was the study adequately powered to detect intervention effects?	If a power calculation was provided and the sample size allowed for detection of intervention effects.	If power calculation was not provided or sample size was inadequate.
ii) Was the randomization procedure adequately described and carried out?	If a random assignment sequence was used and described (computer generated random number tables; use of sealed opaque envelopes.	If the process was not random or adequately described.
iii) Did the study include a control group? (randomized participants not a comparison group)	If the study contained a control group (or usual care group) with randomized participants.	If allocation was not random or there was a treatment.
iv) Did the study present baseline characteristics separately for treatment groups?	If baseline characteristics were presented separately.	If baseline characteristics were not presented separately.
v) Did the study analyses account for potential differences at baseline?	If the statistical procedures accounted for potential differences at baselines e.g. ANOVA or ANCOVA.	If statistical measures did not account for potential differences at baseline.
vi) Were the assessors blinded to treatment allocation at baseline and post-test?	If assessors were blinded to participant allocation at baseline and posttest.	If assessors were not blinded to participant allocation.
vii) Did the study have a dropout of <20% (<6 month follow-up) or <30% (>6 month follow-up) for the primary outcome of weight?	If dropout was <20% for <6 months studies or <30% for studies >6 months.	If dropout was >20% for <6 months studies or >30% for studies >6 months
viii) Did the study use an intention to treat analysis?	If intention to treat analyses was used.	If intention to treat analyses was not used.
ix) Did the study report summary results for each group?	If summary results for each group were presented.	If no summary results were presented.
x) Did the study report precision estimates (e.g., 95% confidence interval) and/or effect sizes?	If precision estimates and/or effect sizes were included.	If precision estimates and/or effect sizes were not included.
