# Supplementary material for: Efficacy of interventions that include diet, aerobic and resistance training components for type 2 diabetes prevention: a systematic review with meta-analysis
Source: Int J Behav Nutr Phys Act. 2014 Jan 15;11:2. doi: 10.1186/1479-5868-11-2 (PMC3898566; doi:10.1186/1479-5868-11-2)
Supplement: Additional file 2 — Characteristics of included studies. [file 1479-5868-11-2-S2.pdf]

Additional file 2. Characteristics of included studies

Author, year and study name	Design	Participants	Intervention groups (n)<br>Intervention Length<br>Follow up	Description of dietary intervention	Description of exercise intervention
**Author**<br>Bersoux et al 2010<br>**SN:** DEAL study	single group pre-post	IFG and/or IGT<br><br>n = 47[#]<br><br>M: n = 21*<br>F: n = 71*<br><br>Age (mean ± SD) = 62 ± 12 years	**Groups**<br>lifestyle INT group only<br>**Intervention**<br>1 yr<br>**Mode**<br>group face to face<br>**Follow up**<br>nil	- weight loss goal of 7%<br>- information sessions on healthful food choices, food labels, portion control, dining out healthy, shopping for healthful foods, decreasing dietary fat, food records and goal setting	- Initial supervised one on one sessions with physical therapist (1 week) to develop exercise program, then self-directed for remainder of study<br>- 150 min PA.wk^{-1}<br>- personalised exercise program that emphasised aerobic exercise<br>- instructed to perform RT for upper extremity, lower extremity and core muscle groups<br>- flexibility exercises included<br>- pedometer: 10000 steps.day^{-1}
**Author**<br>Burtscher et al 2009	two group comparison	IFG<br><br>n = 36[#]<br><br>M: n = 20*<br>F: n = 16*<br><br>Age (mean ± SD) = 57.5 ± 6.9 years	**Groups**<br>counselling only INT n = 18 and counselling + supervised exercise INT n = 18<br>**Intervention**<br>1 yr<br>**Mode**<br>individual face to face<br>**Follow up**<br>nil	**Both groups**<br>- weight loss goal of 5%<br>- achieve diet where total fat intake < 30% and saturated fat intake < 10%	**Both groups**<br>- moderate intensity PA for 30 min.day^{-1}<br>**Counselling + supervised exercise group**<br>- supervised, progressive, individually tailored aerobic exercise program and circuit-type RT sessions for 1 hour two times.wk^{-1} (6-8 strength training exercises with 8-12 repetitions)
**Authors**<br>Lindstrom et al 2003, (Eriksson et al 1999 (Laaksonen et al 2005, Lindstrom et al 2006, Lindstrom et al 2003b, Tuomilehto et al 2001, Uusitupa et al 2003, Uusitupa et al 2000)<br>**SN:** Finnish DPS	RCT	IGT<br><br>n = 434[#]<br><br>M: n = 172*<br>F: n = 350*<br><br>Age (mean ± SD) = 55 ± 7 years	**Groups**<br>lifestyle INT (n = 231) and CON (n = 203, single education session at baseline)<br>**Intervention length**<br>median of 4 yr across the 5 study centres<br>Note: Data for 3 yr used as the 4 yr results were only presented as means.<br>**Mode**<br>individual face to face<br>**Follow up**<br>median of 7 yr<br>Note: data not reported in original article as a specific time-point of follow up.	- BMI goal of < 25 kg.m^{-2} (> 5% weight loss)<br>- diet comprising > 50% carbohydrates, < 30% fat (< 10% saturated fat, < 20% from mono-unsaturated and poly-unsaturated fat), 1 g protein.kg^{-1} ideal body weight<br>- < 300 mg.day^{-1} cholesterol<br>- > 15 g fibre per 1000 kcal<br>- VLCD was implemented if BMI at 6 months still > 30 kg.m^{-2}	- moderate intensity aerobic exercise for at least 30 min.day^{-1}<br>- supervised progressive, individually tailored circuit-type resistance training 2 days.wk^{-1}. Exercises included RT for large muscle groups of the upper and lower body

Author, year and study name	Design	Participants	Intervention groups (n)<br>Intervention Length<br>Follow up	Description of dietary intervention	Description of exercise intervention
**Author**<br>McAuley et al 2002, (Dale et al 2009)	RCT	IGT<br><br>n = 77[#]<br><br>n = 43 completed 2 year follow up (See Note)<br><br>M: n = 26*<br>F: n = 53*<br><br>Age (mean and range) = 43.3 [30-38] years	**Groups**<br>intensive lifestyle INT (n = 25), moderate lifestyle intervention (n = 29) CON (n = 23, 2 week education after 4 month intervention)<br>**Intervention length**<br>4 months<br>**Mode**<br>individual face to face<br>**Follow up**<br>2 yr<br><br>NOTE: intensive and modest INT groups collapsed into single intervention group after 4 month assessments	**Modest program**<br>- diet comprising 50% carbohydrates, 32% fat (11% saturated fat, 14% monounsaturated fat, 7% polyunsaturated fat) and 18% protein<br>- cholesterol 200 mg.day^{-1}<br>- dietary fiber 25 g.day^{-1}<br>**Intensive program**<br>- diet comprising 55% carbohydrates 26% fat (6% saturated fat, 13% monounsaturated fat, 7% polyunsaturated fat) and 18% protein)<br>- cholesterol 140 mg.day^{-1}<br>- dietary fiber 35 g.day^{-1}	**Modest group**<br>- 30 min.day^{-1} of PA for 5 days.wk^{-1}<br>- NB no RT<br>**Intensive group**<br>- individual and group exercise sessions<br>- supervised by exercise consultant (at least once per week)<br>- 20 min.day^{-1} of PA at 80-90% for max heart rate<br>- gym membership to encourage vigorous PA and resistance training at least 2 days.wk^{-1}
**Authors**<br>Page et al 1993 & Page et al 1992	RCT	IGT<br><br>n = 25[#]<br><br>n = 23 completed 2 year follow up<br><br>M: n = 17*<br>F: n = 8*<br><br>Age (mean ± SD) = INT 39 ± 11 years vs CON 40 ± 10 years	**Groups**<br>lifestyle INT groups for the first 6 wk with Diet only, exercise only, diet and exercise. Then all collapsed into Healthy Lifestyle INT group (n = 18), CON (n = 7, placebo pill). Drug group not included in analysis<br>**Intervention Length**<br>6 months<br>**Mode**<br>individual face to face<br>**Follow up**<br>2 yr	- reduction in energy intake (where appropriate) to achieve BMI goal < 25 kg.m-2<br>- diet comprising 50-55% carbohydrates and 30% fat<br>- poly-unsaturated:saturated fat ratio of 1.0<br>- fiber intake 20 g.1000 kcal^{-1}	- 20 min of aerobic exercise for 3 days.wk^{-1} (minimum)<br>- supervised exercise classes provided aerobic and anaerobic exercise, circuit training, swimming and aerobic weight training

Author, year and study name	Design	Participants	Intervention groups (n)<br>Intervention Length<br>Follow up	Description of dietary intervention	Description of exercise intervention
**Author**<br>Payne et al 2008	Quasi-experimental	at risk for diabetes and/or IGT or IFG<br><br>n = 122#<br><br>M: n = 58*<br>F: n = 64*<br><br>Age (mean ± SD) = 52.6 ± 8.6 years	**Groups**<br>lifestyle + gym-based RT INT (n = 62) and lifestyle +home-based RT INT (n = 60)<br>**Intervention length**<br>1 yr<br>**Mode**<br>group face to face<br>**Follow up**<br>nil<br><br>Note: No between groups differences for key diabetes measures. Results are presented in manuscript as combined pre-post results at 1 year	- weight loss goal of >5%<br>- diet comprising < 30% fat and < 10% saturated fat	**Both groups**<br>- ≥ 150 min of weighted PA and ≥ 5 sessions of at least moderate PA.wk^{-1}<br>**Gym-Based RT group**<br>**-** supervised program<br>**-** 2-3 RT sessions.wk^{-1} for 12 wk (wk 7-18 of 52 wk intervention). Sessions of 5 min low intensity aerobic warm-up, 45 min high intensity RT, 5 min of stretching cool down. RT program focused on selected major muscle groups and was incremental<br>**Home-based RT group**<br>**-** unsupervised program<br>**-** 2-3 RT sessions.wk^{-1} for 12 wk (wk 7-18 of 52 wk intervention). Selection of exercises and progressions using body weight exercises and convenient hand-held weights (e.g., cans of food). Wk 13-18, theraband and swiss ball exercises added<br>**- Both groups** were advised to continue the regime from wk 19-52
**Authors**<br>Roumen et al 2008, (Corpeleijn et al 2006, Mensink et al 2003a, Mensink 2003b, Roumen et al 2011)<br>**SN:** The SLIM study	RCT	high risk individuals for IGT<br><br>n = 106#<br><br>M: n = 75*<br>F: n = 72*<br><br>Age (mean ± SD) = INT 54.2 ± 5.8 years vs CON 58.4 ± 6.8 years	**Groups**<br>lifestyle INT (n = 52) and CON (n = 54, brief general advice)<br>**Intervention Length**<br>3 yr<br>**Mode**<br>individual face to face<br>**Follow up**<br>nil	- weight loss goal of 5-7%<br>- diet comprising > 55% of total energy from carbohydrates, < 30-35% fat, < 10% saturated fat, 10-15% protein<br>- fiber > 3 g.MJ^{-1}<br>- cholesterol < 33 mg.MJ^{-1}	- 30 min PA.day^{-1} for at least 5 days.wk^{-1}<br>- access to a supervised exercise program with aerobic and resistance activities for 1 hour.wk^{-1}

Author, year and study name	Design	Participants	Intervention groups (n)<br>Intervention Length<br>Follow up	Description of dietary intervention	Description of exercise intervention
**Authors**<br>Villareal 2006, (Villareal et al 2008, Villareal 2006)	RCT	at risk for diabetes or IGT<br><br>n = 24[#]<br><br>M: n = 9*<br>F: n = 18*<br><br>Age (mean ± SD) = INT 71 ± 2 years vs CON 69 ± 1 years	**Groups**<br>INT (n = 15) and CON (n = 9, instructed to maintain usual dietary and exercise patterns)<br>**Intervention length**<br>26 wk<br>**Mode**<br>group face to face<br>**Follow up**<br>nil	- energy deficit of 750 kcal.day^{-1}<br>- weight loss goal of 10%<br>- diet comprising approximately 50% carbohydrate, 30% fat and 20 % protein	- group exercise supervised training sessions, 3 non-consecutive days.wk^{-1}<br>- 90 min session comprising 15 min warm-up flexibility tasks, 30 min endurance exercise, 30 min strength training and 15 min balance exercises

Authors - Where multiple publications per study are present, the main outcomes paper is listed first, followed by the remaining relevant articles in alphabetical order
Participants – results shown are based on the number of participants reported on in the main outcomes paper (i.e., completed intervention and/or follow up). * Baseline sample (male and female), [#] sample that completed the intervention
CON - control group, **FPG** - Fasting Plasma Glucose, **IFG** - Impaired Fasting Glucose, **IGT** - Impaired Glucose Tolerance, **INT** - intervention group, **mg** - milligram, **MJ** - MegaJoule, **SN** - Study Name, **PA** - Physical Activity, **RCT** - Randomised Controlled Trial, **RT** - Resistance Training, **VLCD** - Very Low Calorie Diet, **wk** - week, **yr** – year, **<** - less than, **>** - greater than
