# Supplementary material for: Efficacy of interventions that include diet, aerobic and resistance training components for type 2 diabetes prevention: a systematic review with meta-analysis
Source: Int J Behav Nutr Phys Act. 2014 Jan 15;11:2. doi: 10.1186/1479-5868-11-2 (PMC3898566; doi:10.1186/1479-5868-11-2)
Supplement: Additional file 3 — Results of included studies. [file 1479-5868-11-2-S3.pdf]

Additional file 3. Results of included studies

Author, year<br>Study Name<br>(SN)	Analysis	Outcome measures	Results for weight and diabetes markers	Results on dietary and exercise outcomes	Retention
**Author**<br>Bersoux et al 2010<br>**SN:** DEAL study	- Mann-Whitney test and Wilcoxon sign rank test for differences between unpaired continuous variables	- weight<br>- FPG<br>- OGTT	**Pre-post results at 6 months**<br>- ↓ weight (pre 89.5 ± 17 kg – post 86.9 ± 16.8 kg, P < 0.001)<br>- FPG did not change (pre 6.05 mmol.L^{-1} – post 6.11 mmol.L^{-1}, no SD, P = 0.14)<br>- 2-h OGTT improved (pre 9.38 mmol.L^{-1} – post 8.44 mmol.L^{-1}, no SD, P = 0.04)	Not reported	- 47/92 (51%) completed 6 month intervention period
**Author**<br>Burtscher et al 2009	- 2-way repeated measures ANOVA for between group differences<br>- Post hoc paired *t*-test with bonferroni correction for within group differences	- weight<br>- FPG<br>- duration of PA/exercise<br>- power output and METs	**Within group results counselling + supervised exercise at 1 yr**<br>- ↓ weight (pre 86.9 ± 13.5 kg – post 84.3 ± 11.5 kg, P < 0.05)<br>- ↓ FPG (pre 5.9 ± 0.4 mmol.L^{-1} – post 5.6 ± 0.4 mmol.L^{-1}, P < 0.05)<br>**Within group results counselling only exercise at 1 yr**<br>- no change in weight (pre 79.4 ± 12.3 kg – post 80.2 ± 12.0 kg, P > 0.05)<br>- ↓ FPG (pre 6.0 ± 0.4 mmol.L^{-1} – post 5.5 ± 0.5 mmol.L^{-1}, P < 0.05)<br>**Between group results at 1 yr**<br>- ↓ weight (P = 0.03)<br>- no difference FPG (P = 0.19)	**Within group results counselling + supervised exercise at 1 yr**<br>no data on within group dietary changes<br>**Exercise**<br>- ↑ power output max (pre 133.5 ± 39.1 W – post 137.5 ± 40.3 W, P < 0.05)<br>- ↑ $METs_{max}$ (pre 6.8 ± 1.2 $METs_{max}$ – post 7.1 ± 1.5 $METs_{max}$, P = 0.01)<br>**Within group results counselling only exercise at 1 yr**<br>No diet or exercise data<br>**Between group results at 1 yr**<br>**Diet**<br>no data on between group dietary changes<br>**Exercise**<br>- ↑ power output max (P = 0.04)<br>- ↑ $METs_{max}$ (P = 0.01)<br>- approximately two-fold ↑ in PA/exercise (counselling + exercise 218 ± 105 min.wk^{-1} vs counselling only 111 ± 166 min.wk^{-1}, P value not shown)	- 36 of 131 (27%) participants completed 1 yr intervention period
**Authors**<br>Lindstrom et al 2003, (Eriksson et al 1999 (Laaksonen et al 2005, Lindstrom et al 2006, Lindstrom et al 2003b, Tuomilehto et al 2001, Uusitupa et al 2003, Uusitupa et al 2000)<br>**SN:** Finnish DPS	- Student's *t*-test for differences at baseline and ANVCOVA adjusted for baseline value	- weight<br>- FPG<br>- OGTT<br>- HBA1C<br>- dietary composition<br>- PA min.wk^{-1}	**Between group differences at 3 yr**<br>**-** ↓ weight (INT -3.5 ± 5.1 kg vs CON -0.9 ± 5.4 kg, P < 0.001)<br>- no change in FPG (INT -0.0 ± 0.7 mmol.L^{-1} vs CON 0.1 ± 0.7 mmol.L^{-1}, P < 0.066)<br>- no change in OGTT (INT -0.5 ± 2.4 mmol.L^{-1} vs CON -0.1 ± 2.2 mmol.L^{-1}, P < 0.066)<br>- ↓ HbA_{1C} (INT -0.2 ± 0.6 mmol.L^{-1} vs CON 0.0 ± 0.6 mmol.L^{-1}, P = 0.002)	**Between group differences at 3 yr**<br>**Diet**<br>- ↓ total energy intake (INT -853 ± 2044 kJ.day^{-1} vs CON -405 ± 1914 kJ.day^{-1}, P = 0.0067)<br>- ↑ carbohydrate %E (INT 3.3 ± 8.0% vs CON 2.0 ± 7.6%, P = 0.007)<br>- ↓ fat %E (INT -4.7 ± 7.7% vs CON -3.2 ± 7.5%, P < 0.001)<br>- ↓ sat fat %E (INT -3.2 ± 4.5% vs CON -1.9 ± 4.9%, P < 0.001)<br>- ↑ fibre (INT 2.4 ± 4.7 g.1000 kcal^{-1} vs CON -1.1 ± 4.1 g.1000 kcal^{-1}, P = 0.001)	- 434/522 (83%) competed 3 yr intervention period

Author, year Study Name (SN)	Analysis	Outcome measures	Results for weight and diabetes markers	Results on dietary and exercise outcomes	Retention
				**Exercise**<br>- No difference in total PA time (INT 50 min.wk^{-1} [-126 to 115 IQR] vs CON 23 min.wk^{-1} [-142 to 171 IQR], P = 0.2415)<br>- ↑ moderate to vigorous leisure time PA (INT 61 min.wk^{-1} [-33 to 168 IQR] vs CON 6 min.wk^{-1} [-91 to 104 IQR], P = 0.0057)	
**Author**<br>McAuley et al 2002, (Dale et al 2009)	- weight changes were assessed using ITT analysis using a mixed model with random effect for person<br>- regression analysis with baseline measure as a covariate or ANCOVA used for other measures.	- weight<br>- FPG<br>- dietary composition<br>- aerobic fitness by predicted VO_{2max}	**Between group results at 4 months**<br>- ↓ weight for both modest INT and intensive INT compared to control (adjusted differences: Modest INT -3.4 kg [-5.4, -1.3; 95% CI, Intensive INT -4.7 kg [-6.9, -2.4; 95% CI], CON pre 102.8 ± 15.3 kg post 101.5 ± 15.1 kg, P = 0.002 and P < 0.001, respectively)<br>- No changes in FPG (adjusted differences: Modest INT -0.1 mmol.L^{-1} [-0.3, 0.2; 95% CI, Intensive INT -4.7 mmol.L^{-1} [-0.4, 0.2; 95% CI], CON pre 5.2 ± 0.6 mmol.L^{-1} post 5.2 ± 0.5 mmol.L^{-1}, P = 0.61 and P = 0.47 respectively)<br>**Between groups results at 2 years**<br>- No differences	**Between group results at 4 months**<br>**Diet**<br>- ↓ total energy intake for modest INT but not sig different for intensive INT (adjusted differences - Modest INT 1705 kJ.day^{-1} [523, 2884; 95% CI], Intensive INT -1012 kJ.day^{-1} [-2215, -192; 95% CI], CON pre 9564 kJ.day^{-1} [no SD or CI] post 8866 kJ.day^{-1} [no SD or CI], P = 0.005, P = 0.098, respectively)<br>- ↑ carbohydrates %E for both INT groups (adjusted differences – Modest INT 7% [3, 11; 95% CI], Intensive INT 5% [1, 9; 95% CI] CON pre 47 ± 6 post 46 ± 8, P = 0.001, P = 0.02, respectively)<br>- ↓ total fat %E for both INT groups (adjusted differences - Modest INT 11% [-15, -7; 95% CI], Intensive INT -9% [-12, -5; 95% CI, CON pre 33 ± 6% post 35 ± 8%, P < 0.001 for both)<br>- ↓ sat fat %E for both INT groups (adjusted differences - Modest INT -7% [-10, -5; 95% CI], Intensive INT -7% [-9, -5; 95% C]I, CON pre 14 ± 3% post 16 ± 5%, P < 0.001 for both)<br>- ↑ protein %E for both INT groups (adjusted differences - Modest INT 4% [1, 6; 95% CI], Intensive INT 3% [1, 6; 95% CI], CON pre 17 ± 4% post 16 ± 3%, P = 0.002, P = 0.01 respectively)<br>- ↑ fiber for intensive INT only (adjusted differences - Modest INT 5.4% [-0.4, 11.2; 95% CI], Intensive INT 8.9% [2.9, 14.9; 95% CI], CON pre 24.5 [no SD] post 21.7 [no SD], P = 0.07, P = 0.004, respectively)<br>**Exercise**<br>- ↑ $VO2_{max}$ for intensive INT only (adjusted differences - Modest INT 0 mL.min^{-1}.kg^{-1} [-3, 3; 95% CI, Intensive INT 4 mL.min^{-1}.kg^{-1} [1, 7; 95% CI], CON pre 29 ± 6 mL.min^{-1}.kg^{-1}	- 77/79 (97 %) completed 4 month intervention period<br>- 62/79 (78 %) completed 2 yr follow up measurements

Author, year Study Name (SN)	Analysis	Outcome measures	Results for weight and diabetes markers	Results on dietary and exercise outcomes	Retention
				post 32 ± 7 $mL.min^{-1}.kg^{-1}$, P = 0.94, P = 0.02, respectively)<br>**Between groups results at 2 years**<br>- No differences	
**Authors**<br>Page et al 1993 & Page et al 1992	- paired and unpaired *t*-tests<br>- Wilcoxon sign rank test<br><br>NOTE: between groups results not reported for relevant outcomes to this review	- BMI/Weight<br>- FPG<br>- diet composition<br>- aerobic fitness by VO_{2max}	**Within group results at 6 months** (n=18)<br>- non-sig reduction in BMI (INT pre 26 ± 4 $kg.m^{-2}$ – post 25 ± 5 $kg.m^{-2}$ vs CON pre 28 ± 4 $kg.m^{-2}$ – 27 ± 4 $kg.m^{-2}$, P value not reported), weight (kg) not reported<br>- no change in FPG (INT pre 5.6 ± 0.6 $mmol.L^{-1}$ – post 5.6 ± 0.7 $mmol.L^{-1}$ vs CON pre 5.8 ± 0.5 $mmol.L^{-1}$ – 5.8 ± 0.5 $mmol.L^{-1}$, P value not reported)<br>**Within group results at 2 yr**<br>- no change in BMI (INT pre 26 ± 4 $kg.m^{-2}$ – post 26 ± 4 $kg.m^{-2}$ vs CON pre 28 ± 4 $kg.m^{-2}$ – 28 ± 4 $kg.m^{-2}$, P value not reported)<br>- ↑ FPG for intervention (INT pre 5.5 ± 0.6 $mmol.L^{-1}$ – post 6.0 ± 1.2 $mmol.L^{-1}$ vs CON pre 5.6 ± 0.5 $mmol.L^{-1}$ – 6.0 ± 0.6 $mmol.L^{-1}$, P < 0.05 and P value not reported, respectively)	**Within group results at 6 months**<br>**Diet**<br>- ↓ total energy intake (INT pre 9455 ± 3925 $kJ.day^{-1}$ – post 7382 ± 2855 $kJ.day^{-1}$ vs CON pre 9288 ± 2370 $kJ.day^{-1}$ – post 8072 ± 1860 $kJ.day^{-1}$, P < 0.01 and P value not reported, respectively)<br>- no change in carbohydrate %E (INT pre 42.4 ± 6.3% – post 44.7 ± 6.7% vs CON pre 42.1 ± 5.8% – post 41.2 ± 4.7, P value not reported)<br>- ↓ fat %E (INT pre 38.8 ± 6.5% – post 33.0 ± 5.4% vs CON pre 37.1 ± 8.4% – post 36.9 ± 8.0%, P < 0.05 and P value not reported, respectively)<br>- ↑ protein %E (INT pre 15.7 ± 3.3% – post 18.5 ± 3.1% vs CON pre 15.6 ± 3.0% – post 16.7 ± 1.9%, P < 0.05 and P value not reported, respectively)<br>- ↑ no change in fiber (INT pre 22.3 ± 8.3 g – post 25.9 ± 10.2 g vs CON pre 24.4 ± 7.3 g – post 22.3 ± 7.0 g, P value not reported)<br>**Exercise**<br>- ↑ VO_{2max} (INT pre 2.4 ± 0.6 $L.min^{-1}$ – post 2.7 ± 0.7 $L.min^{-1}$ vs CON pre 2.6 ± 0.6 $L.min^{-1}$ – post 2.6 ± 0.6 $L.min^{-1}$, P < 0.05 and P value not reported, respectively)<br>**Within group results at 2 yr**<br>- diet and exercise not reported	- 25/31 (81 %) completed 6 month intervention period<br>- 23/31 (74 %) completed 2 yr follow up
**Author**<br>Payne et al 2008	- ITT analysis<br>- repeated measures ANOVA<br>- independent samples *t*-test	- weight<br>- FPG<br>- OGTT<br>- PA<br>- diet composition	**Combined pre-post results at 1 year**<br>- ↓ Weight (-4.07 kg [-4.99, -3.15; 95% CI], P < 0.001)<br>- ↓ FPG (-0.15 $mmol.L^{-1}$ [-0.23, -0.07; 95% CI], P < 0.001)<br>- ↓ 2-h OGTT (-0.34 $mmol.L^{-1}$ [-0.60, -0.08; 95% CI], P < 0.011)	**Combined pre-post results at 1 year**<br>**Diet**<br>- ↓ total energy intake (-1057 $kJ.day^{-1}$ [-0.23, -0.07; 95% CI], P < 0.001)<br>- ↓ fat %E (-2.13 % [-2.96, -1.30; 95% CI], P < 0.001)<br>- ↓ saturated fat %E (-1.43% [-1.88, -0.97; 95% CI], P < 0.001)<br>**Exercise**<br>- ↑ PA (3.09 $sessions.wk^{-1}$ [0.98, 5.19; 95%	- 98/122 (80 %) completed 1 year intervention period

Author, year Study Name (SN)	Analysis	Outcome measures	Results for weight and diabetes markers	Results on dietary and exercise outcomes	Retention
				CI], P = 0.004)<br>- ↑ weighted PA (81.88 $min.wk^{-1}$ [22.93, 140.83; 95% CI], P = 0.007)	
**Authors**<br>Roumen et al 2008, (Corpeleijn et al 2006, Mensink et al 2003a, Mensink 2003b, Roumen et al 2011)<br>**SN:** The SLIM study	- differences within groups tested by student *t*-test.<br>- differences between groups assessed by repeated measures ANOVA for completers analysis and linear mixed models for ITT analysis.	- weight<br>- FPG<br>- OGTT<br>- HBA1C<br>- dietary composition<br>- fitness<br>- PA level	**Between group differences at 3 yr (ITT)**<br>- ↓ weight (INT -1.08 ± 4.30 kg vs CON 0.16 ± 4.91 kg, P = 0.045)<br>- ↑ FPG however the INT was significantly lower (INT 0.32 ± 0.83 $mmol.L^{-1}$ vs CON 0.55 ± 0.82 $mmol.L^{-1}$, P = 0.04)<br>- Small ↓ in 2-h OGTT for INT, though not sig (INT -0.05 ± 2.02 $mmol.L^{-1}$ vs CON 0.89 ± 1.9 $mmol.L^{-1}$, P = 0.086)<br>- no change in HbA_{1C} (INT -0.09 ± 0.43% vs CON -0.10 ± 0.38%, P = 0.838)	**Between group differences at 3 yr**<br>**Diet**<br>- Non-significant ↓ in total energy intake (INT -1100 ± 1900 $kJ.day^{-1}$ vs -300 ± 1800 $kJ.day^{-1}$<br>- ↑ Carbohydrates %E (INT 4.8 ± 5.6% vs - CON 1.2 ± 5.4%, P = 0.001)<br>- ↓ fat %E (INT -4.7 ± 5.9% vs CON -0.5 ± 5.8%, P < 0.001)<br>- ↓ saturated fat %E (INT -2.9 ± 3.2% vs CON -0.7 ± 3.1%, P < 0.001)<br>- ↑ fibre (INT 0.5 ± 0.6 $g.MJ^{-1}$ vs CON 0.2 ± 0.8 $g.MJ^{-1}$, P = 0.05)<br>**Exercise**<br>- ↑ VO_{2max} (INT 0.05 ± 0.35 $L.min^{-1}$ vs CON - 0.6 ± 0.21 $L.min^{-1}$, P = 0.009)	- 106 of 147 (72 %) participants completed 3 yr intervention period
**Authors**<br>Villareal 2006, (Villareal et al 2008, Villareal 2006)	- ITT analysis using last observation carried forward<br>- between group differences assessed using ANOVA with baseline measures as covariates<br>- within group differences assessed by *t*-test for paired samples	- weight<br>- FPG<br>- OGTT<br>- physical performance test, VO_{2peak}, knee extension flexion strength, walking speed over 25 feet	**Between group differences at 26 wk**<br>- ↓ weight (INT -8.2 ± 5.7 kg vs CON 0.7 ± 2.7 kg, P < 0.001)<br>- ↓ FPG (INT -4.4 ± 4% vs CON 4 ± 2%, P < 0.05)<br>- ↓ OGTT (INT -4 ± 12% vs CON 8 ± 4%, P < 0.05)	**Between group differences**<br>**Diet**<br>Not reported<br>**Exercise**<br>- ↑ physical performance test (INT 2.6 ± 2.5% vs CON 0.1 ± 1.0%, P = 0.001)<br>- ↑ VO_{2peak} (INT 1.7 ± 1.6 $mL.min^{-1}.kg^{-1}$ vs CON 0.3 ± 1.1 $mL.min^{-1}.kg^{-1}$, P = 0.02)<br>- ↑ knee extension strength (INT 12.9 ± 12.5% vs CON 4.3 ± 13.0%, P = 0.04)<br>- ↑ Knee flexion strength (INT 25.5 ± 26.6% vs CON 1.1 ± 17.9%, P = 0.008)<br>- ↑ walking speed (INT 7.6 ± 10.2% vs CON - 1.9 ± 12.5%, P = 0.04)	- 24/27 (89 %) completed 24wk intervention period

Outcome measures – data for 1) weight (kg or BMI) 2) diabetic indices including FPG, OGTT, HbA_{1C} 3) measure of dietary change 5) measure of exercise change

Results - For RCTs where between group results are available they are presented. For other study designs within group results are presented. End of intervention and follow up results presented if available. In general for studies with no follow up, results for the last assessment time point are presented. Finnish DPS 3 year results paper is reported on in this table as the 4 year results are only presented as means from a small subset (one study site) of the total study. Page et al did not report between group differences for outcomes relevant to this review, therefore only within group results for relevant outcomes are presented.

CON - control group, **FPG** - Fasting Plasma Glucose, **IFG** - Impaired Fasting Glucose, **IGT** - Impaired Glucose Tolerance, **INT** - intervention group, **IQR** - interquartile range, **ITT** - Intention To Treat, **METs** - Metabolic Equivalents, **mg** - milligram, **sig** - significant, **SN** - Study Name, **PA** - Physical Activity, **RT** - Resistance Training, **VLCD** - Very Low Calorie Diet, **vs** - versus, **wk** - week, **yr** - year, **<** - less than, **>** - greater than, ↑ - increase, ↓ - decrease
